# Safety and Immunogenicity of Poultry Vaccine for Protecting Critically Endangered Avian Species against Highly Pathogenic Avian Influenza Virus, United States

**DOI:** 10.3201/eid3106.241558

**Published:** 2025-06

**Authors:** Todd E. Katzner, Ashleigh V. Blackford, Mary Donahue, Samantha E.J. Gibbs, Julianna Lenoch, Michael Martin, Tonie E. Rocke, J. Jeffrey Root, Darrel Styles, Sunny Cooper, Kristin Dean, Zachary Dvornicky-Raymond, Dominique Keller, Carlos Sanchez, Brett Dunlap, Thomas Grier, Michael P. Jones, Gregory Nitzel, Erin Patrick, Maureen Purcell, Aaron J. Specht, David L. Suarez

**Affiliations:** US Geological Survey, Forest and Rangeland Ecosystem Science Center, Boise, Idaho, USA (T.E. Katzner, M. Purcell); US Fish and Wildlife Service, California Condor Recovery Program, Vero Beach, Florida, USA (A.V. Blackford); US Department of Agriculture, Animal and Plant Health Inspection Service (APHIS) Veterinary Services, Minneapolis, Minnesota, USA (M. Donahue); US Fish and Wildlife Service, National Wildlife Refuge System, Wildlife Health Office, Chiefland, Florida, USA (S.E.J. Gibbs); US Department of Agriculture, APHIS Wildlife Services, National Wildlife Disease Program, Fort Collins, Colorado, USA (J. Lenoch); North Carolina Department of Agriculture and Consumer Services, Raleigh, North Carolina, USA (M. Martin); US Geological Survey, National Wildlife Health Center, Madison, Wisconsin, USA (T.E. Rocke); US Department of Agriculture, APHIS Wildlife Services, National Wildlife Research Center, Fort Collins (J.J. Root); US Department of Agriculture, APHIS Veterinary Services, Riverdale, Maryland, USA (D. Styles); Carolina Raptor Center, Huntersville, North Carolina, USA (S. Cooper, K. Dean); San Diego Zoo Wildlife Alliance, San Diego, California, USA (Z. Dvornicky-Raymond); Los Angeles Zoo and Botanical Garden, Los Angeles, California, USA (D. Keller); Oregon Zoo, Portland, Oregon, USA (C. Sanchez); US Department of Agriculture, APHIS Wildlife Services, Madison, Tennessee, USA (B. Dunlap); Purdue University School of Health Sciences, West Lafayette, Indiana, USA (T. Grier, A.J. Specht); American Eagle Foundation, Kodak, Tennessee, USA (M.P. Jones); Zoetis, Inc., Kalamazoo, Michigan, USA (G. Nitzel); US Department of Agriculture, APHIS Wildlife Services, Knoxville, Tennessee, USA (E. Patrick); US Department of Agriculture, Southeast Poultry Research Laboratory, Agricultural Research Service, Athens, Georgia, USA (D.L. Suarez)

**Keywords:** Influenza, viruses, highly pathogenic avian influenza virus, Black vulture, California condor, Coragyps atratus, Gymnogyps californianus, H5N1, inactivated vaccine, zoonoses, respiratory infections, United States

## Abstract

In 2023, an outbreak of highly pathogenic avian influenza occurred among critically endangered California condors (*Gymnogyps californianus*), and >21 died. We evaluated safety, immunogenicity, vaccination strategies, and correlates of antibody response of an influenza vaccine for poultry in black vultures (*Coragyps atratus*) and then California condors. We noted differences in antibody titers between vaccinated and unvaccinated birds (vultures p<0.004; condors p­<0.02) but no adverse effects of vaccination. All vaccinated vultures and 80% of vaccinated condors showed maximum measured antibody response within the published range associated with survival of vaccinated and virally challenged chickens. We noted weak evidence of higher antibody responses for birds given two 0.5-mL vaccines versus those given one 1-mL vaccine but no correlation between antibody titers and sex for either species or between antibody titers and bone lead concentrations in vultures. Our results prompted initiation of a vaccination program for condors that could reduce spread of this disease among highly threatened species.

Emerging infectious diseases are a growing threat to global biodiversity and particularly to endangered species (*1*). Since 2020, highly pathogenic avian influenza (HPAI) virus (HPAIV) of the goose/Guangdong/1996 (GS/GD/96) lineage, specifically influenza A(H5N1) clade 2.3.4.4b, has caused a panzootic in poultry and wild birds (*2*,*3*) and subsequent spillover into some wild mammals (*4*). Birds, especially those in the orders Anseriformes and Charadriiformes, can be infected with avian influenza viruses, with or without showing clinical signs, and can shed high quantities of virus (*5*), enabling widespread dispersion. HPAIV infections from the current H5N1 lineage were first detected in wild birds in the United States in January 2022 (*6*). In addition, deaths among many wild bird species infected with H5N1 HPAIV appear to have contributed to deaths among predatory and scavenging mammals and birds that consume carcasses of infected animals (*6*–*8*).

One of the highest profile wildlife species known to be affected by HPAI in North America is the California condor (*Gymnogyps californianus*) (Figure 1, panel A). California condors are critically endangered, and only ≈560 birds exist in 5 geographically dispersed wild subpopulations and in captivity (*9*). Exposure to lead, an immunosuppressant that also lethally poisons avian scavengers, has been the most critical factor limiting growth and recovery of condor populations, and the continued existence of this species is largely the result of ongoing field-based and captive conservation efforts that began in the 1970s (*10*). In 2023, the subpopulation of condors that occupies northern Arizona and southern Utah experienced an HPAI outbreak during which >21 birds (≈18% of the subpopulation) died; other subpopulations were not affected (*9*,*11*).

**Figure 1:**
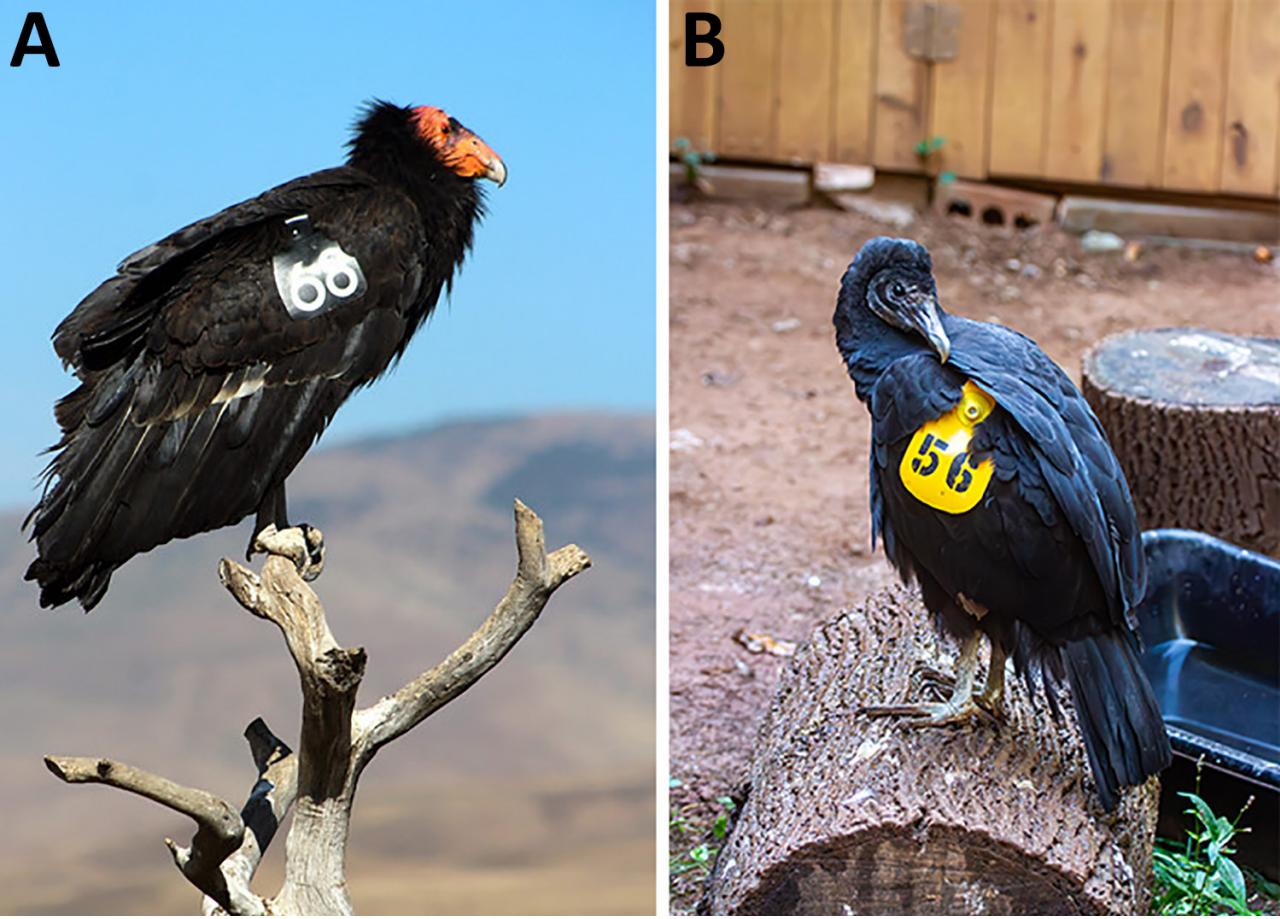
Photographs of avian species tested in study of safety and immunogenicity of poultry vaccine for protecting critically endangered avian species from highly pathogenic avian influenza virus, United States. A) California condor (*Gymnogyps californianus*), whose infection with highly pathogenic avian influenza virus was the motivation for this research. B) Black vulture (*Coragryps atratus*), closely related species used as a surrogate for condors in this research. Photo credits: panel A, US Fish and Wildlife Service public domain; panel B, Todd E. Katzner.

Considering the conservation consequences of that outbreak, vaccination was evaluated as a potential means to reduce illness, death, and virus transmission or shedding (*12*). Vaccines for HPAIV have been used for decades in some countries where the virus is endemic in poultry or where a high likelihood of virus transmission exists in the environment (*13*). However, we found no data on vaccination of condors against avian influenza, although captive studies of other species, including raptors, demonstrated antibody responses (*14*) and a protective immune response (*15*) from administration of an influenza subtype H5N2 vaccine.

For critically endangered species, evaluating the potential risk from vaccination by first conducting trials on surrogate species is appropriate (*16*). Black vultures (*Coragyps atratus*) (Figure 1, panel B) are a good surrogate for California condors because they are susceptible to HPAIV infection (*8*), are co-familial and abundant, and are readily available for study because they are sometimes lethally controlled as a nuisance species (*17*). We evaluated safety, immunogenicity, vaccination regimens, and correlates of antibody response for a conditionally licensed influenza subtype H5N1 vaccine designed for poultry, first in black vultures as a surrogate species, and then in California condors. Our specific objectives were to determine the safety and immunogenicity of the vaccine in vultures and, if warranted by those results, subsequently in condors; to compare the typical prime-boost vaccination regimen used for poultry to a single-vaccination regimen that could be more feasible for wild birds; and to evaluate 2 correlates of antibody response of vultures, sex and lead exposure.

## Materials and Methods

### Vaccines

For our trials, we used an H5N1 subtype, reverse genetics-derived, inactivated vaccine that includes genes from the gyrfalcon/2014 virus, avian influenza vaccine (1057.R1 serial 590088) (rgH5N1) (Zoetis, Inc., https://www.zoetis.com), as previously described (*18*). The US Department of Agriculture (USDA) (*19*), the US Fish and Wildlife Service (USFWS), the North Carolina Department of Agriculture, and the North Carolina Wildlife Resources Commission specifically authorized use of that conditionally licensed vaccine for vultures, and numerous state and federal agencies subsequently approved its use in condors (Appendix Table 1).

### Lead Exposure of Vultures

We evaluated lead exposure of vultures by measuring bone lead concentrations with a portable x-ray fluorescence (XRF) device (*20*) 66 days after they were removed from the wild and 53 days after the start of the vaccine trial. An XRF device measures backscatter radiation signals to noninvasively infer lead density in bone, a technique used for years in humans and recently adapted for use with birds of prey (*21*,*22*). We placed the XRF device against the leg (tarsometatarsus) of the living bird, where it measured spectra that were then interpreted as lead concentrations (*21*). Bone lead measurements are typically considered indicative of cumulative or long-term exposure to lead, whereas blood measurements are indicative of more recent, acute, exposure (*23*). Because all condors in the study were captive and fed untainted food, we did not evaluate their bone lead.

### Study Design

We randomly assigned 28 vultures to 1 of 3 treatment groups (Appendix). We vaccinated 1 group of 10 vultures (4 male, 6 female) with a prime-boost (2-vaccine) regimen typically used for poultry, in which 0.5 mL is given on day 0 and again on day 21. We gave another group of 10 vultures (all female) a 1-vaccine regimen of 1.0 mL on day 0. A third group of 8 vultures (1 male, 7 female) remained unvaccinated to serve as a control group. 

We administered vaccine subcutaneously between the shoulder blades because that location is near the standard subcutaneous vaccine site used for poultry (back of the neck) and because it avoids the cervical and other air sacs along the back. We did not use a sham vaccine (e.g., saline) on control birds. Experienced wildlife husbandry professionals monitored all birds daily for signs of lethargy, reduction of food intake, and other potential indicators of an adverse vaccine reaction. They also visually evaluated the vaccine site 3 days after each vaccination. We collected blood samples from all vultures 1 day before and 10, 21, 31, and 42 days after the initial vaccination. We used 42 days postvaccination (dpv) as the experimental endpoint because that is the standard for vaccine experiments in poultry (*18*), and because of challenges in keeping vultures in captivity.

We used a similar design for the condor trial, giving the 2-vaccine regimen to 10 birds and the 1-vaccine regimen to 10 birds and using 5 unvaccinated birds as controls (Appendix). However, because condors are highly endangered and no single facility holds large numbers, we made several modifications to the design of the trial to reduce risk and stress to birds. Specifically, because birds are held at multiple different locations, we identified 10 birds at the Los Angeles Zoo and Botanical Gardens (LAZ) (2 male, 6 female) and San Diego Zoo Wildlife Alliance (SDZ) (1 male, 1 female) for the prime-boost regimen, 10 at SDZ (4 male, 2 female) and Oregon Zoo (ORZ) (2 male, 2 female) for the single-vaccine regimen, and 5 at ORZ (3 male, 2 female) as controls. We staged implementation of the trial and vaccinated 3 condors in each regimen group first, then vaccinated the remaining condors in that group only after confirming that the first birds had no apparent short-term negative effects, as described for vultures. For condors, we administered the vaccine in the inguinal region to avoid the cervical air sacs, which are larger than those found in vultures. Finally, because of concerns for potential problems caused by frequent handling of these critically endangered birds, we conducted blood draws for serologic analyses less frequently than for vultures, at 0, 21, and 42 dpv.

### Laboratory Analyses

We evaluated vaccine-induced antibody formation for all birds by using both an ELISA, AI MultiS-Screen Ab Test (IDEXX Laboratories, https://www.idexx.com), to detect antibodies to the nucleoprotein of influenza A viruses and a hemagglutination inhibition (HI) assay (*24*). We also evaluated blood chemistry for vultures at 31 dpv (Appendix).

### Data Analyses

We used HI titers to statistically compare the antibody response among treatment groups. We inferred potential conservation value of vaccinating condors by comparing HI titers obtained in our study to a prior study that established correlates of vaccine protection by linking postvaccination HI titers to survival of domestic chickens (*Gallus gallus domesticus*) in a viral challenge (*18*). Chickens given the same vaccine used in our study had antibody titers ranging from 16 to 1,024, and 100% survived infection with the virus (*18*). Thus, to interpret the conservation value of the vaccines, we conservatively assumed that an antibody titer of >16 was responsive to vaccination and that a titer of >32 likely provided adequate protection against death. We considered HI titers <8 as nonresponsive and likely nonprotective.

We used a Wilcoxon test to compare antibody titers for vaccinated and unvaccinated birds using wilcox.test in R (The R Project for Statistical Computing, https://www.r-project.org). We used Kruskal-Wallis tests (kruskal.test) with Dunn’s test (package dunn.test) of multiple comparisons in R to evaluate differences in antibody titers and in blood chemistry for birds given 0, 1, or 2 vaccinations. We used another Wilcoxon test to test for sex-specific differences in antibody responses to vaccination for the subset of groups in which both sexes were represented. We generated effect estimates for both the Kruskal-Wallis and Wilcoxon tests (*25*). Finally, because lead can suppress immune response to vaccination, we used a Pearson correlation (corr.test in R) to evaluate HI titers of vultures as a function of bone lead concentration. In all cases, we considered p<0.05 indicative of differences between groups.

## Results

### Vaccine Safety

Vaccinated vultures showed no adverse effects, and we detected no changes in behavior or food intake. We did detect small (≈5 mm) and temporary nodules at the injection site on 2 vultures after the first round of vaccination. Blood chemistry of vaccinated vultures was generally unremarkable (Appendix Table 2). No condors vaccinated in this trial showed adverse behavioral or physical effects linked to vaccination.

### Vaccine Immunogenicity in Vultures

**Figure 2:**
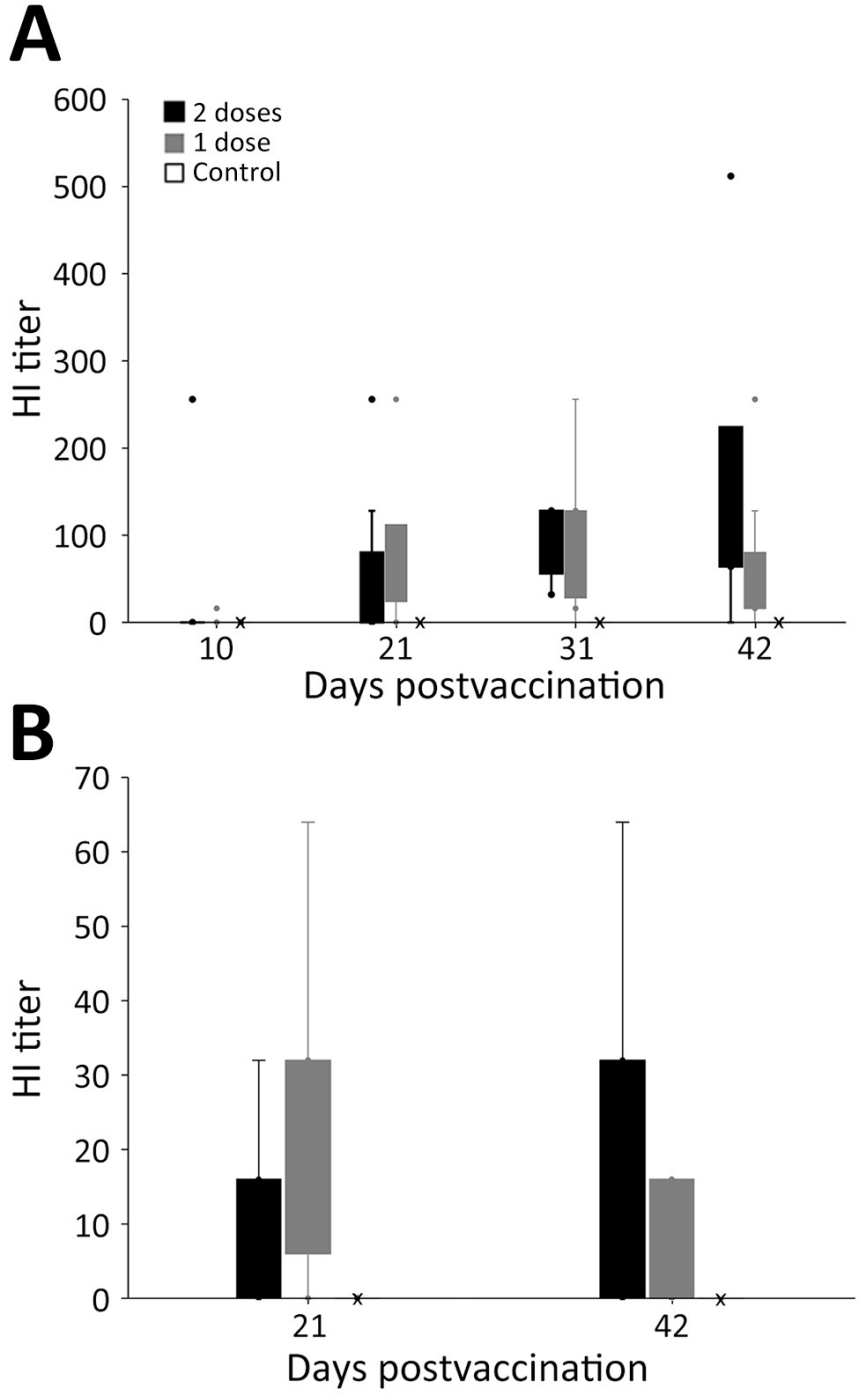
Hemagglutination inhibition (HI) titers in a study of safety and immunogenicity of poultry vaccine for protecting critically endangered avian species from highly pathogenic avian influenza virus, United States. A) Titers for 28 black vultures (*Coragryps atratus*); B) titers for 25 California condors (*Gymnogyps californianus*). Birds were included in 1 of 3 highly pathogenic avian influenza vaccine trial groups comprised of 10 birds given two 0.5-mL vaccinations at days 0 and 21, another 10 birds given a single 1-mL vaccination at day 0, and 8 vultures and 5 condors that were unvaccinated negative controls. Vaccinated animals were given a 1057.R1 serial 590088 avian influenza vaccine, H5N1 subtype, reverse genetics-derived, inactivated vaccine (see main text for details on the vaccine). For vultures, postvaccination blood draws were conducted at 10, 21, 31, and 42 days after first vaccination; for condors, blood draws were on days 21 and 42. Box tops and bottoms show quartiles, whiskers are 95% CI, dots outliers; X indicates 0 values for control groups.

Of the 28 vultures, 27 had negative ELISA results at the start of the study; 1 bird had a positive ELISA result before vaccination, indicating prior exposure to an avian influenza A virus (Appendix Table 3). We detected a positive HI antibody response in all 20 vaccinated vultures at some point during the 42-day trial and in none of the control (unvaccinated) birds (Table 1; Figure 2, panel A; Appendix Table 3). Statistical tests suggested that HI titers were different, and higher, for vaccinated birds than for unvaccinated birds starting at 21 days after the first vaccination: at 10 dpv, Wilcoxon rank-sum value (*W*) = 81 (p = 0.962); at 21 dpv, *W* = 28 (p = 0.004); at 31 dpv, *W* = 4 (p<0.001); at 42 dpv, *W* = 8 (p<0.001). Outcomes of statistical tests were similar when we excluded the 1 bird previously exposed to an influenza A virus (Appendix).

**Table 1:** Hemagglutination inhibition antibody titers in surrogate and target species in a study of safety and immunogenicity of poultry vaccine for protecting critically endangered avian species from highly pathogenic avian influenza virus, United States*

Time, dpv				χ^2^	p value	*η^2^*	Intergroup difference (p value)		
	HI titer [SD] (range) per vaccine regimen						None–1 vaccine	None–2 vaccines	1–2 vaccines
	None, n = 8	1-vaccine, n = 10	2-vaccine, n = 10						
Black vultures (*Coragryps atratus*)									
10	0 [0.7] (0–2)	0 [5.1] (0–16)	0 [81.0] (0–256)	0.01	0.994	−0.08	NA	NA	NA
21	0	32 [96.8] (0–256)	16 [82.9] (0–256)	9.65	0.008	0.31	−3.09 (**0.006**)	−2.019 (0.130)	1.13 (0.774)
31	0	96 [75.6] (0–256)	64 [40.5] (32–128)	16.01	<0.001	0.56	−3.50 (**0.001**)	−3.55 (**0.001**)	−0.06 (1.00)
42	0	64 [73.6] (0–256)	128 [180.5] (0–512)	16.26	<0.001	0.57	−2.71 (**0.020**)	−3.99 (**<0.001**)	−1.35 (0.528)
California condors (*Gymnogyps californianus*)									
21	0	16 [19.3] (0–64)	4 [11.0] (0–32)	7.49	0.024	0.25	−2.72 (**0.020**)	−1.59 (0.334)	1.38 (0.501)
42†	0	12 [7.9] (0–16)	32 [20.3] (0–64)	7.42	0.025	0.25	−1.40 (0.484)	−2.71 (**0.0203**)	−1.23 (0.656)

*Bold text indicates statistical significance. Birds were vaccinated with a 1057.R1 serial 590088 Avian Influenza Vaccine, H5N1 subtype, reverse genetics-derived, inactivated vaccine. See main text for details on the vaccine and vaccination regimens. Titers were compared by using Kruskal-Wallis test reporting a χ^2^, a p value, and an effect estimate (*η^2^*); degrees of freedom = 2 in all tests and, when different, a Dunn’s multiple comparison (reporting a Z statistic and a p value). dpv, days postvaccination; HI, hemagglutination inhibition; NA, no difference detected so multiple comparison not relevant. †For day 42, HI titers are only available for 6 condors given a single vaccination.

Of 20 vaccinated birds, 19 (95%) had titers >32 on >1 postvaccination blood draw. The exception was 1 of the birds in the 1-vaccine group; its highest titer was 16 on day 42. The bird with presumed prior influenza A virus exposure was in the 2-vaccine group and appeared to have a faster and generally stronger antibody response to vaccination than the other birds (Appendix Table 3). At 10 dpv, 2 (10%) birds had titers >16; at 21 dpv, 13 (65%) birds had titers above that level, 19 (95%) did at 31 dpv, and 18 (90%) did at 42 dpv.

All vaccinated vultures also tested positive for an antibody response by ELISA at some point over the 42-day trial. At 42 dpv, 1 bird that received the 2-vaccine regimen and 1 bird that received the 1-vaccine regimen had negative ELISA results; HI titers measured at the same time were 0 for the bird in the 2-vaccine group and 256 for the bird in the 1-vaccine group.

### Vaccine Immunogenicity in Condors

We detected a positive HI antibody response in 16 (80%) of the 20 vaccinated condors at some point during the 42-day trial and in none of the control (unvaccinated) birds (Table 1; Figure 2, panel B; Appendix Table 4). Of the 4 nonresponsive but immunized birds, 1 that received the 1-vaccine regimen had an HI titer of 0; the other 3 birds, 2 from the 1-vaccine group and 1 from the 2-vaccine group, had HI titers of 8. Two of the birds that showed no antibody response from the 1-vaccine group were only tested for HI antibodies at 21 dpv and not at 42 dpv. Statistical tests suggested higher HI titers at both blood draws for vaccinated birds relative to unvaccinated birds: at 21 dpv, *W* = 17.5 (p = 0.020); at 42 dpv, *W* = 12.5 (p = 0.017) (note that at 42 dpv, HI data were available for only 6 birds in the 1-vaccine group). Nine (45%) of 20 vaccinated birds had HI titers >32 on >1 of the postvaccination blood draws. At 21 dpv, 10 (50%) birds had an HI titer >16, and 4 of those also were >32. At 42 dpv, 10 (62.5%) of 16 birds had an antibody response of >16, and 6 of those also were >32.

Of the 20 vaccinated condors, 18 (90%) also tested positive for an antibody response by ELISA at some point over the 42-day trial. At 21 dpv, 9 birds given the 1-vaccine regimen and 4 given the 2-vaccine regimen had a positive ELISA result. At 42 dpv, four 1-vaccine regimen birds and seven 2-vaccine regimen birds had positive ELISA test results. The 2 birds with a negative ELISA were both in the 2-vaccine group, and both had HI titers of 16 at day 21 and of 0 at day 42.

### Response to Vaccine Regimen

We detected statistically relevant differences between vultures that were vaccinated and those that were not but not between vultures that received the different vaccination regimens (Table 1). The mean maximum HI titer (188.8) of vultures that received the 2-vaccine regimen trended higher compared with birds that received the 1-vaccine regimen (mean maximum = 126.4). At 21 dpv, 80% of the birds in the 1-vaccine group and 50% in 2-vaccine group had titers >32. At 32 dpv, 80% of the birds in the 1-vaccine group and 100% in the 2-vaccine group had titers >32. At 42 dpv, 70% of the birds in the 1-vaccine group and 90% in the 2-vaccine group had titers >32.

For condors, we detected statistically relevant differences between vaccinated and unvaccinated birds, but not between those given the different vaccine regimens (Table 1). Despite that finding, at 21 dpv, only 1 of the 2-vaccine regimen birds had an HI titer >32, and 4 had titers >16; three birds in the 1-vaccine group had titers >32 and 6 had titers >16. At 42 dpv, of the birds in the 2-vaccine group, 6 had titers >32, and 7 had titers >16; of the birds given the 1-vaccine regimen, 0 had titers >32 and 3 had titers >16 (note that HI data are only available for 6 birds in the 1-vaccine group at 42 dpv). In the 2-vaccine group, only 1 bird was deemed nonresponsive over the course of the trial (titer was 8); in the 1-vaccine group, 3 birds met that criterion (titers were 0, 8, and 8).

### Correlates of Antibody Response

We detected no sex-related differences in antibody response for vultures given the 2-vaccine regimen (Table 2) or condors given either regimen (Table 3). Although the sex ratio of our sample group was not even, for vultures, the maximum antibody response in each group was always highest for female birds. That did not appear to be the case for condors.

**Table 2:** Antibody titers and bone lead levels in black vultures (*Coragryps atratus*) in a trial to evaluate safety and immunogenicity of poultry vaccine for protecting a critically endangered avian species from highly pathogenic avian influenza virus, United States*

Birds	Mean lead	10 dpv			21 dpv			31 dpv			42 dpv	
		Mean HI [SD]	Median HI (range)		Mean HI [SD]	Median HI (range)		Mean HI [SD]	Median HI (range)		Mean HI [SD]	Median HI (range)
Sex												
F, n = 6	22.76	43 [104]	0 (0–256)		80 [99]	48 (0–256)		101 [46]	128 (32–128)		235 [218]	128 (64–512)
M, n = 4	18.67	0	0		8 [16]	0 (0–32)		56 [16]	64 (32–64)		96 [64]	128 (0–128)
*W*		14			15			12			6.5	
p value		0.54			0.56			1.00			0.25	
*R*		0.37			0.39			0.35			0.25	

*Antibody titers (as determined by hemagglutination inhibition) and mean bone lead levels (as determined by XRF), organized by sex for black vultures given a 1057.R1 serial 590088 avian influenza vaccine, H5N1 subtype, reverse genetics-derived, inactivated vaccine (see main text for details on the vaccine). Only vultures given a 2-vaccine regimen were considered; those given a 1-vaccine regimen were all female. Titers were compared between sexes at each timepoint with a Wilcoxon rank-sum test (*W*), a p value (p), and an effect estimate (*R*). dpv, days postvaccination; HI, hemagglutination inhibition.

**Table 3:** Antibody titers for California condors (*Gymnogyps californianus*) in a study of the safety and immunogenicity of poultry vaccine for protecting critically endangered avian species from highly pathogenic avian influenza virus, United States*

Vaccine regimen	21 dpv			42 dpv	
	Mean HI [SD]	Median HI (range)		Mean HI [SD]	Median HI (range)
1-vaccine†					
Sex					
F, n = 7	32 [23]	24 (16–64)		16 [0]	16 (16–16)
M, n = 3	11 [12]	8 (0–32)		6 [8]	4 (0–16)
*W*	20.5			7	
p value	0.08			0.21	
*R*	0.45			0.26	
2-vaccine					
Sex					
F, n = 4	8 [8]	8 (0–16)		21 [24]	16 (0–64)
M, n = 6	11 [18]	0 (0–32)		32 [0]	32 (32–32)
* W*	11			6	
p value	1.00			0.32	
* R*	0.33			0.24	

*Antibody titers were determined by hemagglutination inhibition organized by sex for California condors given a 1057.R1 serial 590088 Avian Influenza Vaccine, H5N1 subtype, reverse genetics-derived, inactivated vaccine (see main text for details on the vaccine and vaccination regimen). HI titers were measured only at 21 and 42 dpv. Titers were compared between sexes at each timepoint with a Wilcoxon rank-sum test (*W*), a p value (p), and an effect estimate (*R*). dpv, days postvaccination; HI, hemagglutination inhibition. †For day 42 , HI titers are only available for 2 female and 4 male condors given a single vaccination.

We did not detect a relationship between bone lead concentrations and antibody response of vultures at any time postvaccination. The absolute value of the correlation coefficients tended to be low, ranging from 0.1 to 0.5, and the tests indicated no evidence of correlations (p>0.05) (Appendix Table 5). However, 7 of the 8 correlation coefficients were negative, and the strongest antibody responses tended to be in the vultures with the lowest bone lead concentrations (Appendix Figure).

## Discussion

Many types of HPAIV vaccines have been developed (*26*), including inactivated whole virus vaccines, subunit vaccines, and live vectored viral vaccines (*27*). Risk analysis for this vaccination trial included consideration of the potential to stimulate a protective immune response, legal availability of the vaccine, and the antigenic relatedness of vaccines to a potential field challenge. That approach led us to select the inactivated adjuvanted reverse genetics vaccine for this trial because it safely stimulated immune responses in multiple avian species and because it has ≈95.6% amino acid similarity to the currently circulating H5N1 2.3.4.4b virus isolates.

Although nearly all birds of both species responded immunologically to the vaccine, the generally stronger short-term antibody responses of black vultures compared with California condors are notable. Interspecific differences are not surprising because vaccines developed for one species can have unexpected effects in other species and related species can have substantially different responses to vaccination (*28*). Despite those differences, maximum HI titers of vultures given a 2-vaccine regimen were similar to those reported for domestic fowl given a similar vaccination regimen (i.e., vulture maximum titers were 32–512, chicken titers at 42 dpv were 16–1,024) (18). Similarly, maximum titers of condors given a 2-vaccine regimen were 8–64, and all but 1 titer was within the lower end of the range reported for chickens.

Work with endangered species presents many hurdles and often precludes the possibility of testing the effectiveness of a vaccine with a viral challenge (*29*). Furthermore, because of the dramatic impacts of HPAI on wild condors, we conducted the trials rapidly and in an extremely urgent context. Together with biosafety considerations, those issues made it impractical to conduct a logistically difficult viral challenge for the vultures or to evaluate longer-term immune response. However, we can draw inference from prior work with this vaccine. As we noted, 100% of chickens given this same vaccine responded with similar antibody levels and survived a viral challenge at 42 dpv (*18*). Given the similarity of those responses, had a viral challenge at 42 dpv had been feasible, we reason that most if not all vultures, and perhaps condors, would likely have survived.

The vaccine we evaluated was developed for prime-boost (2-vaccine) application. However, because trapping and handling can stress condors, we evaluated a 1-vaccine regimen as an alternative to the originally designed regimen. Despite the lack of a statistical difference between antibody responses associated with the 2 regimens, qualitative evaluation suggested that antibody responses were weaker and dissipated more rapidly for the birds that received the 1-vaccine regimen. Thus, we suspect that if birds were given a viral challenge, birds that received prime and boost vaccinations would have been more effectively protected than those vaccinated once. However, because our study ended at 42 dpv, we could not evaluate the potential for differences in waning immunity (*30*) between the 2 vaccine strategies.

Our failure to detect a statistical effect of either sex or bone lead concentration on antibody response might have been because of the small sample sizes and skewed sex ratios in our trials. Trends in the data suggested that if our sample size had been larger and the sex ratios more even, we might have detected differences in responses between sexes. Likewise, in the case of the response to bone lead concentrations, the statistical approach we used might not have uncovered difficult-to-detect patterns. We noted that, regardless of vaccine regimen, the birds with the strongest antibody response were also those with the lowest bone lead level. Therefore, subsequent trials might evaluate the presence of threshold-type effects in those responses.

Given the virulence and spread of the currently circulating HPAIV, vaccination of threatened and endangered wild birds could be a potential tool to mitigate losses from this disease. Vaccination may be particularly relevant when the resiliency of populations has decreased to the extent that naturally occurring illness and death from disease could impair the species’ long-term persistence. Despite the potential value of that approach, negative consequences of vaccination are possible, and this trial and implementation in the condor program are unique within the United States. Given the importance of economic considerations associated with poultry farming, close coordination with the USDA and many other federal and state agencies was essential to receive authorization to implement these vaccination trials. However, the outcomes from these trials were positive enough, and the threat from HPAIV so great, that the USFWS subsequently decided to initiate a vaccination program for the California Condor Recovery Program (*11*). By October 2024, a total of 207 condors had received >1 vaccination (*30*).

Species-specific variations in physiological response to vaccination are characteristic problems associated with vaccination programs for wildlife (*31*). Despite such variations, evidence suggests that vaccination strategies that reach <50% of an affected wildlife population can still be effective at staving off extinction (*32*,*33*). Those trends, together with results of our work, suggest several next steps for protecting endangered wildlife, whether condors or other species, from infectious diseases. Vaccination of the at-risk population of the target species can begin once safety, immunogenicity, and vaccination regimens have been established and some correlate of protection established, either from published work with other species or from direct challenge trials. Critical next steps include monitoring vaccine effectiveness in field settings and demographic modeling to understand the most effective strategy for vaccination of wild animals. Specifically, given the challenges inherent in vaccinating wild animals, using life history traits of the species in question, together with population modeling, can confirm the costs and benefits of vaccination in relation to its risks and, thus, can help identify vaccination strategies that can stabilize populations and enable them to recover. Relevant vaccination strategies might involve varying the time of year (especially relative to reproductive seasons and seasonal variation in survivorship), age classes, and proportion of the population that is vaccinated. 

In summary, we evaluated safety, immunogenicity, vaccination regimens, and correlates of antibody response for a conditionally licensed influenza subtype H5N1 vaccine designed for poultry in black vultures and California condors. Our work suggests that the use of licensed vaccines can be a realistic strategy to aid in conservation of condors and potentially other species facing similar threats, especially those with small and highly threatened populations.

Appendix Additional information on safety and immunogenicity of poultry vaccine for protecting critically endangered avian species from highly pathogenic avian influenza virus, United States.
