# Supplementary material for: Safety and Immunogenicity of Poultry Vaccine for Protecting Critically Endangered Avian Species against Highly Pathogenic Avian Influenza Virus, United States
Source: Emerg Infect Dis. 2025 Jun;31(6):1131–9. doi: 10.3201/eid3106.241558 (PMC12123928; doi:10.3201/eid3106.241558)
Supplement: Appendix — Additional information on safety and immunogenicity of poultry vaccine for protecting critically endangered avian species from highly pathogenic avian influenza virus, United States. [file 24-1558-Techapp-s1.pdf]

Article DOI: https://doi.org/10.3201/eid3106.241558

EID cannot ensure accessibility for supplementary materials supplied by authors. Readers who have difficulty accessing supplementary content should contact the authors for assistance.

Safety and Immunogenicity of Poultry Vaccine for Protecting Critically Endangered Avian Species from Highly Pathogenic Avian Influenza Virus, United States

Appendix

Supplemental Methods and Results

Selection of Surrogate Species

The California condor and other American vultures are in the Family Cathartidae (Order Cathartiformes) and are related to, but distinct from, vultures in the Order Accipitriformes that occupy Eurasia and Africa (*1*,*2*). California condors are a monotypic species and thus no congener was available as a surrogate. Although their closest relatives are the Andean condor (*Vultur gryphus*) and the king vulture (*Sarcoramphus papa*), both are of conservation concern (*3*) and neither is available for trials. As such, black vultures were chosen because they are abundant, not of conservation concern, available for trials, and reasonably closely related to condors.

We set a target of 30 birds for our trials, 10 in each of the two treatment groups and in the control group. We were able to obtain 28 wild vultures for the trials. Given the critically endangered status of the condors, we were only able to access 25 birds for trials and we did not use sham vaccinations. In both cases we included 10 birds in each treatment group and put the remainder in the control group.

Experimental Animals

During routine control activities undertaken by the U.S. Department of Agriculture (USDA), 28 black vultures were captured on 02 May 2023 in Stewart County, TN, USA. The birds were transferred the following day to the Carolina Raptor Center (Charlotte, NC, USA), and placed in quarantine. As of the date of capture, HPAIV had previously been detected in Tennessee, including in black vultures (*4*).

Captive vultures were held in groups of 4–8 individuals in specially designed housing permitted by the U.S. Fish and Wildlife Service (USFWS). Husbandry and housing were at standards of care applied in zoological and wildlife rehabilitation settings (*5*). All birds were fed a diet of commercially available rats (*Rattus norvegicus domestica*) or young chickens (*Gallus gallus domesticus*) both from RodentPro (Inglefield, IN, USA), or stillborn hogs (*Sus scrofa*) from a commercial production facility. Plumage characteristics indicated that these birds were composed of a mixture of both adult and pre-adult individuals. Sexes of all birds were determined by comparing genetic sequences for CDH-W and CDH-Z genes (*6*,*7*) at Veterinary Molecular Diagnostics, Inc. (Milford, OH, USA).

California condors used in this trial were from the captive population held at the San Diego Zoo Wildlife Alliance (SDZ; n = 8), the Los Angeles Zoo and Botanical Garden (LAZ; n = 8) and the Oregon Zoo (ORZ; n = 9). We chose for this trial birds that were healthy, represented a wide range of ages, and that were reasonable for use in the trial (not breeding, feasible to capture, etc.). All birds were maintained in standard zoo housing in groups of 1–6 individuals at these facilities and provided diets of carrion (typically domestic rabbits [*Oryctolagus cuniculus*], goats [*Capra hircus*], or cattle [*Bos taurus*]). Because condor populations are small and the birds intensively managed, age (1–16 years old) and sex of each individual bird is known.

Collection, transport, and holding of vultures and condors were completed with state and federal permits from wildlife management and veterinary authorities, Certificates of Veterinary Inspection, and with approvals from the USDA, and a U.S. Geological Survey (USGS) Institutional Animal Care and Use Committee (IACUC). Because of the large number of permits and approvals required for this study, we report them in Appendix Table 1.

Analyses

The ELISA was a blocking or competitive assay that can detect nucleoprotein antibodies to any influenza A viruses, while the HI assay is targeted only to antibodies of the H5 subtype. The antigen used for the HI assay was the GyrFalcon/WA/41088–6/2014 H5N8 which matches the vaccine antigen (*8*). The HI assay endpoint titer was the highest dilution that gave a detectable response in the assay and is reported as the reciprocal of that dilution (e.g., 1:32 is reported as 32). We used a cutoff value of optical density (OD) <0.5 to identify positive ELISA test results.

Because one bird had previously been exposed to an influenza A virus, we tested the impact on statistical significance by re-running our initial statistical tests on datasets without that bird. As we did not detect statistical differences between birds vaccinated once vs twice, there was no value to re-running statistical tests on the dataset without that bird to compare outcomes from different levels of vaccination (i.e., HI titers appeared slightly lower for birds given one vaccination, previously exposed bird was vaccinated twice and its removal from one dataset would have simply made the two datasets more similar).

At 31 dpv we drew additional blood from vultures for chemistry analyses conducted at a commercial laboratory (Moichor Inc., San Francisco, CA, USA). Because of the large number of statistical tests that we ran, we interpreted conservatively the results of statistical analyses of blood chemistry data.

Results

Black vultures were first vaccinated on 16 May 2023, ≈7 weeks after the first condor was found dead in the field.

Outcomes (i.e., statistical significance) from tests on the dataset without the bird previously exposed to an influenza A virus were identical to those conducted on the dataset with that bird (10 dpv: $W = 81$, $p = 0.599$; 21 dpv: $W = 28$, $p = 0.005$; 31 dpv: $W = 4$, $p<0.001$; 42 dpv: $W = 8$, $p<0.001$).

Leukocyte counts, and levels of total protein, globulin, phosphorous, and lactate dehydrogenase (LDH) were higher for birds given a two-vaccine regimen than for unvaccinated (control) birds. We detected no other potentially relevant differences in blood chemistry for vaccinated vs unvaccinated birds, and there were no blood chemistry parameters for which we detected differences between birds the one- versus two-vaccine regimen.

Condors were vaccinated July–September 2023.

Any use of trade, firm, or product names is for descriptive purposes only and does not imply endorsement by the US government. All opinions expressed in this paper are the authors and do not necessarily reflect the policies and views of the USDA. The findings and conclusions in this article are those of the authors and do not necessarily represent the views of the US Fish and Wildlife Service. This paper has been peer reviewed and approved for publication consistent with USGS Fundamental Science Practices (https://pubs.usgs.gov/circ/1367).

References

1. Johnson JA, Brown JW, Fuchs J, Mindell DP. Multi-locus phylogenetic inference among New World vultures (Aves: Cathartidae). Mol Phylogenet Evol. 2016;105:193–9. PubMed https://doi.org/10.1016/j.ympev.2016.08.025

2. Mindell DP, Fuchs J, Johnson JA. Phylogeny, taxonomy, and geographic diversity of diurnal raptors: Falconiformes, Accipitriformes, and Cathartiformes. In: Sarasola JH, Grande JM, Negro JJ. Birds of prey: biology and conservation in the XXI century. Cham (Switzerland): Springer; 2018. p. 3–32.

3. International Union for the Conservation of Nature (IUCN). The IUCN red list of threatened species, version 2024-1 [cited 2025- Feb 5]. https://www.iucnredlist.org

4. US Department of Agriculture. Detections of Highly Pathogenic Avian Influenza in Wild Birds, 2024 [cited 2024 Aug 5]. https://www.aphis.usda.gov/livestock-poultry-disease/avian/avian-influenza/hpai-detections/wild-birds

5. Arent L. Raptors in captivity: guidelines for care and management. Blaine (WA): Hancock House Publishers Ltd.; 2007.

6. Ito H, Sudo-Yamaji A, Abe M, Murase T, Tsubota T. Sex identification by alternative polymerase chain reaction methods in Falconiformes. Zoolog Sci. 2003;20:339–44. PubMed https://doi.org/10.2108/zsj.20.339

7. Chang HW, Chou TC, Gu DL, Cheng CA, Chang CC, Yao CT, et al. An improved PCR method for gender identification of eagles. Mol Cell Probes. 2008;22:184–8. PubMed https://doi.org/10.1016/j.mcp.2007.12.004

8. Spackman E, Suarez DL, Lee CW, Pantin-Jackwood MJ, Lee SA, Youk S, et al. Efficacy of inactivated and RNA particle vaccines against a North American Clade 2.3.4.4b H5 highly pathogenic avian influenza virus in chickens. Vaccine. 2023;41:7369–76. PubMed https://doi.org/10.1016/j.vaccine.2023.10.070

Appendix Table 1. Permits and approvals obtained for study of safety and immunogenicity of a reverse genetics killed vaccine developed for poultry when used in wild black vultures (*Coragyps atratus*) and California condors (*Gymnogyps californianus*).

Purpose	Granting agency and permit or protocol number
Capture of vultures	USFWS #MB018937
Export of vultures from TN	TN Official Interstate Health Certificates # 508959, 508960, and 508961<br>TWRA Scientific Collection Permit #5880
Import of vultures into NC	NC Department of Agriculture Authorizations # W23–00059, W23–00060, W23–00061<br>NCWRC Wildlife Collection License #23-SC01559
Holding and vaccination of vultures in NC	NCWRC Captivity License for Holding #23-CP00254<br>USFWS Scientific Collecting Permit #MB72348B<br>NC Department of Agriculture Authorization for holding (not numbered)<br>USDA Letter of Authorization to USFWS
Holding of condors at zoos - federal	SDZ: TE8210B-0<br>LAZ - USFWS: TE58866B-0<br>ORZ - USFWS: ES077388
Holding of condors at zoos - state	SDZ: # 1730<br>LAZ - CDFW: 2081a-2014–052–00
IACUC for research for condors and vultures	Protocol #2023–002 from USGS Forest and Rangeland Ecosystem Science Center
Transport of samples from vaccinated individuals	USDA veterinary permit for import and transport of controlled materials, organisms and vectors #44372

*CDFW, California Department of Fish and Wildlife; IACUC, Institutional Animal Care and Use Committee; LAZ, Los Angeles Zoo and Botanical Garden; NC, North Carolina; NCWRC, North Carolina Wildlife Resources Commission; ORZ, Oregon Zoo; SDZ, San Diego Zoo Safari Park; TN, Tennessee; TWRA, Tennessee Wildlife Resources Agency; USDA, US Department of Agriculture; USFWS, US Fish and Wildlife Service; USGS, US Geological Survey.

Appendix Table 2. Blood chemistry values at 31 d postvaccination for black vultures given no vaccine, a 1-vaccine regimen, or a 2-vaccine regimen*

	Vaccine regimen			χ^2	p		Intergroup differences (p value)		
Parameter	None	1-Vaccine	2-Vaccine	stat	value	η^2	none - one	none - two	one - two
Leukocytes	13,640 [3,564]	17,173 [7,108]	20,400 [7,101]	7.73	0.021	0.23	−2.22 (0.08)	−2.63 (**0.03**)	−0.44 (1.00)
Heterophils	8,040 [2,860]	9,899 [5,737]	10,478 [4,746]	4.23	0.120	0.09	NA	NA	NA
Lymphocytes	3,722 [1,601]	5,511 [1,939]	3,880 [3,242]	2.86	0.239	0.03	NA	NA	NA
Monocytes	293 [407]	523 [647]	1,180 [1,500]	5.41	0.067	0.14	NA	NA	NA
Basophils	160 [162]	309 [186]	149 [274]	5.21	0.074	0.13	NA	NA	NA
Eosinophils	328 [838]	305 [1,558]	1,560 [2,336]	3.47	0.176	0.06	NA	NA	NA
Heterophil, %	60.25 [13.74]	62.50 [14.66]	65.70 [17.80]	0.54	0.763	−0.06	NA	NA	NA
Lymphocytes, %	35.75 [11.21]	29.35 [8.72]	19.85 [12.43]	3.19	0.203	0.05	NA	NA	NA
Monocyte, %	3.30 [2.39]	3.25 [3.52]	7.05 [4.48]	4.46	0.107	0.10	NA	NA	NA
Basophil, %	1.30 [1.03]	1.85 [0.71]	0.95 [1.07]	3.04	0.219	0.04	NA	NA	NA
Eosinophil, %	3.25 [5.43]	1.90 [7.75]	6.70 [6.92]	1.85	0.397	−0.01	NA	NA	NA
Total Protein	3.65 [0.31]	4.00 [0.61]	4.40 [0.33]	9.57	0.008	0.33	−1.54 (0.37)	−3.09 (**0.006**)	−1.59 (0.33)
Albumen	1.65 [0.14]	1.60 [0.12]	1.60 [0.19]	4.86	0.088	0.12	NA	NA	NA
Globulin	2.00 [0.23]	2.40 [0.61]	2.60 [0.35]	11.21	0.004	0.40	−2.06 (0.12)	−3.33 (**0.003**)	−1.31 (0.57)
AST	32.50 [4.58]	32.00 [11.26]	34.00 [7.37]	0.37	0.830	−0.07	NA	NA	NA
Uric acid	5.10 [1.4]	3.80 [1.89]	5.00 [1.83]	3.04	0.219	0.05	NA	NA	NA
BUN	3.00 [0.83]	2.00 [0.00]	2.50 [1.13]	7.32	0.026	0.24	2.48 (**0.04**)	0.34 (1.00)	−2.13 (0.10)
Calcium	9.45 [0.53]	9.5 [0.48]	9.9 [0.57]	2.52	0.284	0.02	NA		
Phosphorus	0.95 [0.36]	1.30 [0.70]	1.90 [0.38]	9.67	0.008	0.33	−2.18 (0.09)	−3.04 (**0.007**)	−0.88 (1.00)
Sodium	146.70 [7.55]	148.00 [4.36]	145.20 [5.87]	0.009	0.996	−0.09	NA	NA	NA
Potassium	2.05 [0.52]	1.90 [0.40]	2.20 [0.42]	0.67	0.715	−0.06	NA	NA	NA
Chloride	111.30 [5.96]	110.40 [4.93]	109.80 [6.24	0.85	0.654	−0.05	NA	NA	NA
Cholesterol	206.50 [25.01]	228.00 [43.58]	240.00 [50.97]	1.21	0.547	−0.03	NA	NA	NA
CK	804 [403]	1,036 [230]	1,006 [320]	1.66	0.437	−0.01	NA	NA	NA
LDH	491 [118]	600 [228]	716 [336]	7.071	0.029	0.22	−1.15 (0.75)	−2.64 (**0.02**)	−1.54 (0.37)

*Vaccine regimens consisted of no vaccine (control), a 1-vaccine regimen (1.0 mL), or a 2-vaccine regimen (0.5 mL each) of an inactivated 1057.R1 serial 590088 Avian Influenza Vaccine, H5N1 subtype (see main text for details on the vaccine and vaccination regimens). Blood draws were measured at 31 d postvaccination. Values were compared with a Kruskal-Wallis test and a p value (p), and an effect estimate (η2); degrees of freedom = 2 in all tests) and, when different, a Dunn's Multiple Comparison (reporting a Z statistic and a p-value). NA, not applicable because no difference detected so multiple comparison not relevant. Bold font indicates statistical significance ($p<0.05$). Decimals are reported when SD <100. AST, aspartate aminotransferase; BUN, blood urea nitrogen; CK, creatinine kinase; LDH, lactate dehydrogenase.

Appendix Table 3. Hemagglutination inhibition endpoint titers and ELISA optical density cutoff values for 28 black vultures, each in one of three highly pathogenic avian influenza vaccine trial groups*

		Hemagglutination inhibition endpoint titer, dpv				ELISA OD, dpv		
Bird ID	Vaccine regimen	10	21	31	42	21	31	42
25346	2-vaccine	0	128	64	128	<0.5	<0.5	<0.5
25347	2-vaccine	256	256	128	512	<0.5	<0.5	<0.5
25348	2-vaccine	0	64	128	128	<0.5	<0.5	<0.5
25349	2-vaccine	0	0	32	0	<0.5	<0.5	>0.5
25350	2-vaccine	0	0	128	64	<0.5	<0.5	<0.5
25351	2-vaccine	0	0	32	64	>0.5	>0.5	<0.5
25352	2-vaccine	0	0	64	128	<0.5	<0.5	<0.5
25353	2-vaccine	0	0	64	128	<0.5	<0.5	<0.5
25354	2-vaccine	0	32	64	128	<0.5	<0.5	<0.5
25355	2-vaccine	0	32	128	512	<0.5	<0.5	<0.5
25356	1-vaccine	0	32	128	64	<0.5	<0.5	<0.5
25357	1-vaccine	0	256	256	128	<0.5	<0.5	<0.5
25358	1-vaccine	0	32	128	256	<0.5	<0.5	>0.5
25359	1-vaccine	0	64	64	64	<0.5	<0.5	<0.5
25360	1-vaccine	0	64	128	0	<0.5	<0.5	ND
25361	1-vaccine	0	32	16	16	<0.5	<0.5	<0.5
25362	1-vaccine	0	0	0	16	<0.5	<0.5	<0.5
25363	1-vaccine	0	0	32	64	<0.5	<0.5	<0.5
25364	1-vaccine	0	32	64	64	<0.5	<0.5	<0.5
25365	1-vaccine	16	256	128	64	<0.5	<0.5	<0.5
25366	0 vaccines	0	0	0	0	>0.5	>0.5	>0.5
25367	0 vaccines	0	0	0	0	>0.5	>0.5	>0.5
25368	0 vaccines	0	0	0	0	>0.5	>0.5	>0.5
25369	0 vaccines	2	0	0	0	>0.5	>0.5	>0.5
25370	0 vaccines	0	0	0	0	>0.5	>0.5	>0.5
25371	0 vaccines	0	0	0	0	>0.5	>0.5	>0.5
25374	0 vaccines	0	0	0	0	>0.5	>0.5	>0.5
25376	0 vaccines	0	0	0	0	>0.5	>0.5	>0.5

*Endpoint titer is the highest dilution that gave a detectable response in the assay, reported as the reciprocal value of that dilution. OD values <0.5 were considered a positive reaction. Birds were given a 1057.R1 serial 590088 Avian Influenza Vaccine, H5N1 subtype, reverse genetics-derived, inactivated vaccine (see main text for details on the vaccine and vaccination regimen). Trial groups were comprised of 10 birds given 2 0.5-mL vaccinations at days 0 and 21, 10 birds given a single 1-mL vaccination at day 0, and 8 birds that were unvaccinated negative controls. Post-vaccination blood draws were conducted at 10, 21, 31, and 42 d after first vaccination. dpv, days post-vaccination; ID, identification; OD, optical density.

Appendix Table 4. Hemagglutination inhibition (HI) endpoint titers and ELISA optical density (OD) cutoff values for 25 California condors, each in one of three highly pathogenic avian influenza (HPAI) vaccine trial groups.

		Hemagglutination inhibition endpoint titer, dpv		ELISA OD, dpv	
Bird ID	Vaccine regimen	21	42	21	42
992482-2X	2-vaccine	0	16	>0.5	<0.5
993545-2X	2-vaccine	0	64	>0.5	<0.5
995515-2X	2-vaccine	0	32	>0.5	<0.5
995497	2-vaccine	16	0	>0.5	>0.5
995517	2-vaccine	8	0	<0.5	>0.5
995532	2-vaccine	16	32	<0.5	<0.5
995631	2-vaccine	32	32	<0.5	<0.5
995511	2-vaccine	16	0	>0.5	>0.5
4003959	2-vaccine	0	32	<0.5	<0.5
4004280	2-vaccine	0	32	>0.5	<0.5
4003960	1-vaccine	0	16	<0.5	<0.5
4003966	1-vaccine	16	16	<0.5	>0.5
4004235	1-vaccine	0	0	<0.5	>0.5
4004256	1-vaccine	64	16	<0.5	>0.5
4003807	1-vaccine	32	0	<0.5	<0.5
4004234	1-vaccine	16	8	<0.5	>0.5
B10216 #42 8-24	1-vaccine	8	ND	<0.5	>0.5
B70051 #869 8-24	1-vaccine	8	ND	>0.5	<0.5
B70164 #544 8-24	1-vaccine	16	ND	<0.5	>0.5
C10008 #1065 8-24	1-vaccine	32	ND	<0.5	<0.5
A60020 #154	0 vaccines	0	0	>0.5	ND
A80242 #121	0 vaccines	0	0	>0.5	ND
B10217 #432	0 vaccines	0	0	>0.5	ND
B80020 #99	0 vaccines	0	0	>0.5	ND
C30005 #1071	0 vaccines	0	0	>0.5	ND

*Endpoint titer is the highest dilution that gave a detectable response in the assay, reported as the reciprocal value of that dilution. OD values <0.5 were considered a positive reaction. Birds were given a 1057.R1 serial 590088 Avian Influenza Vaccine, H5N1 subtype, reverse genetics-derived, inactivated vaccine (see main text for details on the vaccine and vaccination regimen). Trial groups were comprised of 10 birds given 2 0.5-mL vaccinations at days 0 and 21, 10 birds given a single 1-mL vaccination at day 0, and 5 that were unvaccinated negative controls. Postvaccination blood draws were conducted on days 21 and 42. dpv, days postvaccination; ID, identification; ND, no data; OD, optical density.

Appendix Table 5. Pearson correlation between bone lead concentrations and antibody response for vultures given a 1-vaccine regimen (n = 10 birds) or a 2-vaccine regimen (n = 10 birds)

No. vaccinations	DPV	*R*	*t*	*df*	p value
1	10	−0.113	−0.323	8	0.755
	21	−0.364	−1.104	8	0.302
	31	−0.278	−0.819	8	0.437
	42	−0.349	−1.054	8	0.323
2	10	−0.408	−1.264	8	0.242
	21	−0.507	−1.662	8	0.135
	31	0.246	0.718	8	0.493
	42	−0.408	−1.262	8	0.242

*Birds were vaccinated with A1057.R1 serial 590088 Avian Influenza Vaccine, H5N1 subtype, reverse genetics-derived, inactivated vaccine was administered. See main text for details on the vaccine and vaccination regimens. Also shown are results for a significance test for correlation, a test statistic, and degrees of freedom. *df*, degrees of freedom; DPV, days postvaccination; *R*, Pearson correlation; *t*, test statistic.

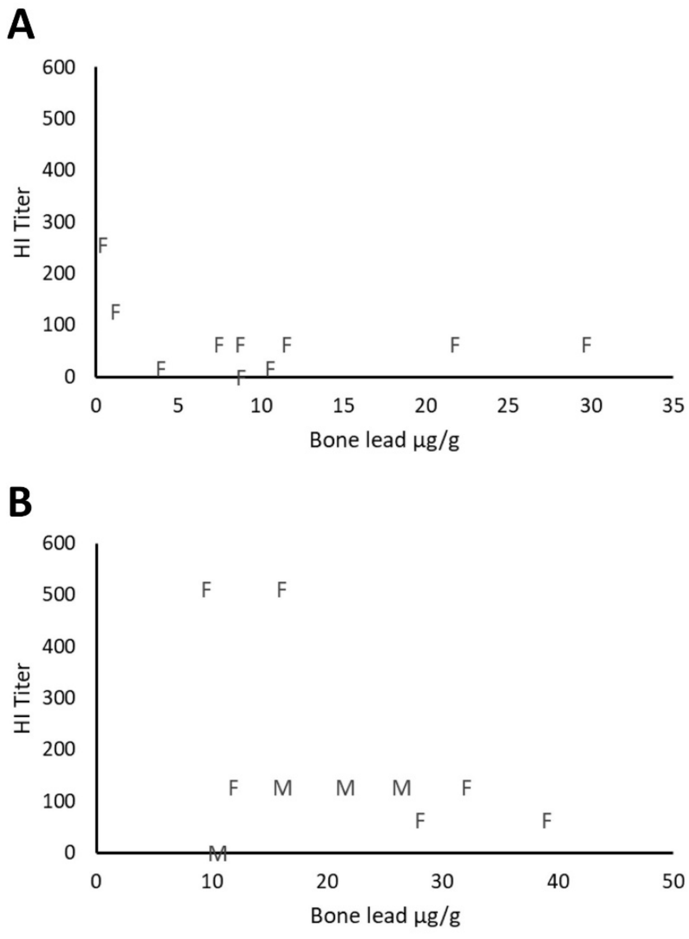

Appendix Figure. Relationship between bone lead concentrations (measured 53 dpv) and antibody response (hemagglutination inhibition titers at 42 dpv) for black vultures given a 1-vaccine (A) and 2-vaccine (B) regimen of a 1057.R1 serial 590088 Avian Influenza Vaccine, H5N1 subtype, reverse genetics-derived, inactivated vaccine (see main text for details on the vaccine and vaccination regimens). Sex of the birds is indicated by the symbols used, female (F) or male (M). dpv, days postvaccination; HI, hemagglutination inhibition.
